# Focal myocardial fibrosis detected with magnetic resonance late Gadolinium enhancement imaging and diffuse interstitial fibrosis determined with histological staining of endomyocardial biopsy specimens in patients with non-ischemic left ventricular systolic dysfunction: two distinct entities?

**DOI:** 10.1186/1532-429X-11-S1-P17

**Published:** 2009-01-28

**Authors:** Simon Schalla, Sebastiaan Bekkers, Robert Dennert, Robert J van Suylen, Johannes Waltenberger, Tim Leiner, Stephane Heymans

**Affiliations:** University Hospital Maastricht, Maastricht, Netherlands

**Keywords:** Late Gadolinium Enhancement, Left Ventricular Dysfunction, Endomyocardial Biopsy, Left Ventricular Systolic Dysfunction, Ventricular Systolic Dysfunction

## Introduction

Focal myocardial fibrosis detected with magnetic resonance late gadolinium enhancement imaging (LGE) as well as diffuse interstitial fibrosis seen on endomyocardial biopsy specimens are associated with an adverse prognosis in patients with non-ischemic left ventricular dysfunction.

## Purpose

We tested the hypothesis if these two forms of fibrosis are linked to each other and whether focal fibrosis becomes visible if a certain amount of diffuse fibrosis is present. Thus, the aim of this study was to determine whether focal fibrosis detected non-invasively with LGE is related to the degree of diffuse fibrosis seen on histologic specimens obtained from endomyocardial biopsy in patients with idiopathic cardiomyopathy.

## Methods and results

Fifty-five patients (30 males, 25 females, age 47 ± 14 years, range 18 – 73 years) with impaired systolic function on intitial echocardiography underwent magnetic resonance cine and LGE imaging (1.5 T Gyroscan Intera, Philips Medical Systems, Best, The Netherlands) and endomyocardial biopsy. The scanparameters were 1) steady-state free precession sequence for cine imaging with slice thickness 6 mm, slice gap 4 mm, TR/TE 3.8/1.9 ms, flip angle 50°, FOV 350 mm, matrix 256 × 256, 22–25 phases per cardiac cycle, 2) Look-Locker sequence to determine the inversion time for the subsequent late enhancement scan to optimally ''null'' left ventricular myocardium (typical range 200–280 ms) with slice thickness 10 mm, TR/TE 3.8/1.9 ms, flip angle 8°, FOV 370 mm, resolution 256 × 256, 39 phases, phase interval 20–21 ms and 3) multislice T1-weighted 3D inversion-recovery gradient-echo sequence for LGE imaging (10 minutes after intravenous administration of 0.2 mmol/kg Gd-DTPA with an injection rate of 3 ml/sec) with slice thickness 12 mm, slice gap 6 mm, TR/TE 4.2/1.3 ms, flip angle 15°, FOV 400 mm, resolution 256 × 256.

The mean LVEF determined with cine MR-imaging was 37 ± 13%, EDV 234 ± 122 ml and LV massa 135 ± 47 g. Twenty patients had LGE, the majority of patients did not show focal fibrosis on MR imaging. On biopsies, the mean collagen volume fraction was 6.2 ± 5.3%. No correlation was found between LGE and histology. Furthermore, no correlation was found between the grade of fibrosis on histological examination and LVEDV or LV mass.

## Conclusion

Focal fibrosis was uncommon in this group of patients with impaired systolic function. No correlation was found between diffuse and focal fibrosis and, thus, focal fibrosis as seen with LGE is not related to a manifestation of a certain degree of diffuse interstitial fibrosis. The underlying pathophysiologic process for the development of focal fibrosis remains to be investigated.

